# Convergent genetic aberrations in murine and human T lineage acute lymphoblastic leukemias

**DOI:** 10.1371/journal.pgen.1008168

**Published:** 2019-06-14

**Authors:** Benjamin J. Huang, Anica M. Wandler, Lauren K. Meyer, Monique Dail, Anneleen Daemen, Deepak Sampath, Qing Li, Xinyue Wang, Jasmine C. Wong, Joy Nakitandwe, James R. Downing, Jinghui Zhang, Barry S. Taylor, Kevin Shannon

**Affiliations:** 1 Department of Pediatrics, University of California San Francisco, San Francisco, CA, United States of America; 2 Department of Oncology Biomarker Development, Genentech, South San Francisco, CA, United States of America; 3 Department of Bioinformatics & Computational Biology, Genentech, South San Francisco, CA, United States of America; 4 Department of Translational Oncology, Genentech, South San Francisco, CA, United States of America; 5 Division of Hematology/Oncology, Department of Medicine, University of Michigan, Ann Arbor, MI, United States of America; 6 Department of Pathology, St Jude Children’s Research Hospital, Memphis, Tennessee, United States of America; 7 Department of Computational Biology, St Jude Children’s Research Hospital, Memphis, Tennessee, United States of America; 8 Marie-Josée and Henry R. Kravis Center for Molecular Oncology, Memorial Sloan Kettering Cancer Center, New York, NY, United States of America; 9 Human Oncology and Pathogenesis Program, Memorial Sloan Kettering Cancer Center, New York, NY, United States of America; 10 Department of Epidemiology and Biostatistics, Memorial Sloan Kettering Cancer Center, New York, NY, United States of America; 11 Helen Diller Family Comprehensive Cancer Center, University of California San Francisco, San Francisco, CA, United States of America; Seattle Children's Research Institute, UNITED STATES

## Abstract

The lack of predictive preclinical models is a fundamental barrier to translating knowledge about the molecular pathogenesis of cancer into improved therapies. Insertional mutagenesis (IM) in mice is a robust strategy for generating malignancies that recapitulate the extensive inter- and intra-tumoral genetic heterogeneity found in advanced human cancers. While the central role of "driver" viral insertions in IM models that aberrantly increase the expression of proto-oncogenes or disrupt tumor suppressors has been appreciated for many years, the contributions of cooperating somatic mutations and large chromosomal alterations to tumorigenesis are largely unknown. Integrated genomic studies of T lineage acute lymphoblastic leukemias (T-ALLs) generated by IM in wild-type (WT) and *Kras* mutant mice reveal frequent point mutations and other recurrent non-insertional genetic alterations that also occur in human T-ALL. These somatic mutations are sensitive and specific markers for defining clonal dynamics and identifying candidate resistance mechanisms in leukemias that relapse after an initial therapeutic response. Primary cancers initiated by IM and resistant clones that emerge during *in vivo* treatment close key gaps in existing preclinical models, and are robust platforms for investigating the efficacy of new therapies and for elucidating how drug exposure shapes tumor evolution and patterns of resistance.

## Author summary

A lack of predictive cancer models is a major bottleneck for prioritizing new anti-cancer drugs for clinical trials. We comprehensively profiled a panel of primary mouse T lineage leukemias initiated by insertional mutagenesis and found remarkable similarities with human T-ALL in regard to overall mutational burden, the occurrence of specific somatic mutations and large chromosomal alterations, and concordant gene expression signatures. We observed frequent duplication of the *Kras* oncogene with loss of the normal allele, which has potential therapeutic implications that merit further investigation in human leukemia and in other preclinical models. Mutations identified in mouse leukemias that relapsed after *in vivo* treatment with signal transduction inhibitors were also observed in relapsed human T-ALL, indicating that this model system can be utilized to investigate strategies for overcoming intrinsic and acquired drug resistance. Finally, preclinical models similar to the one described here that are characterized by a normal endogenous tumor microenvironment and intact immune system will become increasingly important for testing immunotherapy approaches for human cancer.

## Introduction

Most new anti-cancer agents fail in the clinic [1]. Whereas cancer cell lines have been integral to the development of most anti-cancer drugs and exhibit genotype-specific responses to some targeted inhibitors, they fail to model many fundamental properties of primary tumors. Patient derived xenograft (PDX) models are promising platforms for testing anti-cancer drugs, but also have inherent limitations, including lack of an intact immune system or normal tumor microenvironment and failure to fully recapitulate the clonal heterogeneity of advanced human cancers [1–3]. Mouse models in which cancers arise due to mutations introduced into the germline or following exposure to chemical mutagens also have distinct advantages and liabilities. Genetically engineered mouse (GEM) models reproduce many morphologic features of advanced human cancers, but diverge substantially with respect to the overall mutational burden and pattern of copy number alterations [4–8]. Whereas models initiated by chemical carcinogens may more accurately reflect the mutational profile of some human cancers [4, 6], extensive genetic variability and the inability to perform technical replicates are substantial barriers to widespread preclinical use.

IM was developed as an unbiased strategy for cancer gene discovery [9]. This approach initially involved injecting mice with retroviruses, which integrated into the genome and promoted tumorigenesis by activating proto-oncogenes or disrupting tumor suppressors. The locations of exogenous viral DNA insertions also served as molecular sequence tags that facilitated identifying candidate cancer genes. An appealing aspect of IM models is that they recapitulate the inter- and intra-tumoral genetic heterogeneity that is a hallmark of advanced human cancers [2]. Furthermore, technical advances such as the *PiggyBAC* and *Sleeping Beauty* transposon systems both expanded the spectrum of cancers that can be modeled and yielded tumors with insertions within many genes that are altered in corresponding human cancers [10–17].

We previously generated primary T-ALLs by injecting C57Bl/6 x 129Sv/Jae F1 mouse pups harboring a conditional mutant *Kras* oncogene *(Lox-STOP-Lox Kras*^*G12D*^*)* and congenic WT animals with the MOL4070LTR retrovirus [18]. Subsequent activation of endogenous *Kras*^*G12D*^ expression both accelerated disease onset and increased penetrance. Importantly, this system differs from other GEM models of *RAS*-driven cancers because the *Kras*^*G12D*^ mutation is a secondary event that cooperates with antecedent retroviral integrations [19, 20], and thus reflects the most common pathogenic sequence in patients [21]. T-ALLs initiated by IM typically exhibited 3–6 dominant clonal retroviral integrations, many of which occurred within or adjacent to known cancer genes. Interestingly, most of these leukemias also acquired somatic *Notch1* mutations, which are highly prevalent in human T-ALLs [19] and have also been observed in other murine models of T-ALL [22–24]. This unexpected observation raised the possibility that non-insertional genetic mechanisms broadly contribute to tumorigenesis in cancer models driven by IM. In this study, we set out to more thoroughly define the genomic and transcriptional landscape of a cancer model initiated by IM and to assess the contribution that non-insertional genetic mechanisms have on tumorigenesis and clonal evolution upon treatment with signal transduction inhibitors.

## Results

### The mutational landscape of IM-induced T-ALL is similar to that of human T-ALL

**Fig 1:**
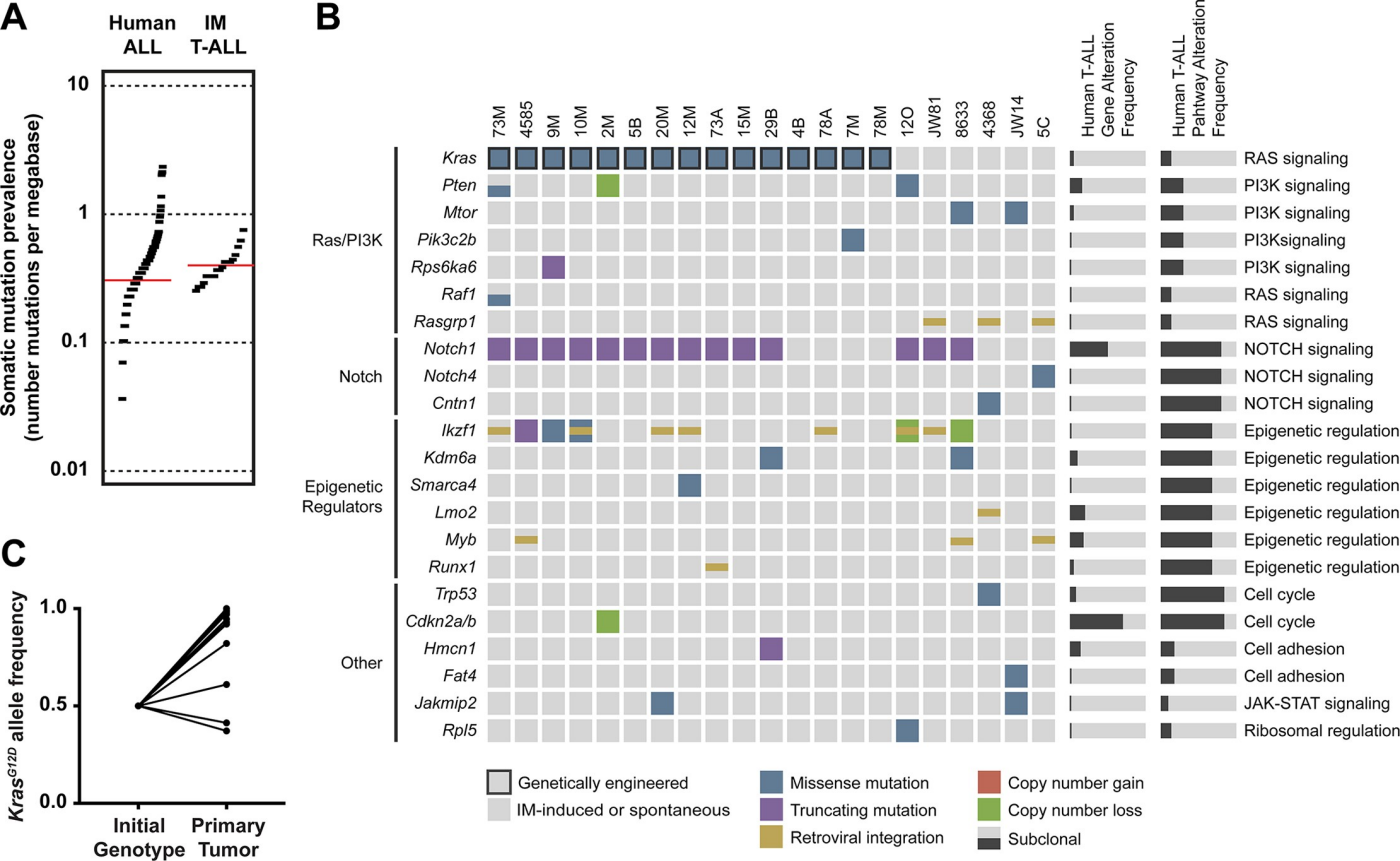
Somatic alterations and retroviral integrations in IM-induced T-ALLs recapitulate features of human T-ALL. (**A**) The overall mutational burden of IM-induced T-ALLs is similar to human ALL. (**B**) SNVs and indels in high-likelihood T-ALL driver genes are frequent and recurrent in IM-induced T-ALLs. Pathways that are most frequently affected include Ras, PI3K, Notch, Ikaros, and transcriptional regulators. Corresponding gene and pathway alterations are recurrent in human T-ALL as reported by Liu, *et al*. (**C**) Frequent copy neutral loss of heterozygosity occurs at the *Kras* locus with duplication of the mutant *Kras*^*G12D*^ allele in murine T-ALLs.

We performed whole exome sequencing (WES) on 21 independent IM-induced T-ALLs that were tested in preclinical efficacy studies of MEK and PI3 kinase (PI3K) inhibitors [20]. These leukemias included 15 primary T-ALLs isolated from *Kras*^*G12D*^ mice and 6 from WT mice (hereafter referred to as *Kras*^*WT*^). The mutational burden in T-ALLs induced by IM ranged from 0.2 to 0.8 somatic alterations per megabase of exome sequence (**Fig 1A**), which is similar to the mutation burden reported in human ALL [25].

WES identified all 14 known *Notch1* mutations [19], and also uncovered somatic mutations in the Notch pathway genes *Notch4* and *Cntn1* in two additional leukemias (**Fig 1B**). Thus, the frequency of somatic mutations in genes linked to Notch signaling (16 of 21; 75%) was similar to what is observed in human T-ALL (**Fig 1B**), with *NOTCH1*/*Notch1* targeted most frequently in both species.

*IKZF1* encodes the Ikaros transcription factor that functions as a regulator of lymphocyte differentiation. Ten independent T-ALLs harbored a variety of *Ikzk1* alterations including retroviral integrations predicted to disrupt the coding region (n = 7), missense or truncating mutations (n = 3), and copy number losses (n = 2) (**Fig 1B**). Five additional genes that are mutated in human T-ALL and broadly regulate gene transcription or epigenetic programming were altered by retroviral integration *(Myb*, *Lmo2*, *Runx1)* or mutation (*Kdm6a*, *Smarca4*). *Myb* integrations and *Kdm6a* mutations were observed in three and two independent leukemias, respectively. Overall, 14 of 21 T-ALLs had one or more alterations of genes classified as transcriptional/epigenetic regulators, which is similar to the frequency observed in human T-ALL (**Fig 1B**). However, the involvement of specific genes varied between species. For example, while *Ikzf1* was affected far more often in murine T-ALL, *IKZF1* is recurrently mutated or deleted in relapsed human ALL and in certain high-risk leukemia subtypes at diagnosis [26–28].

Somatic Ras/PI3K pathway alterations were detected in 6 of 6 *Kras*^*WT*^ leukemias (100%), but in only 4 of 15 *Kras*^*G12D*^ T-ALLs (27%). *Rasgrp1*, which encodes a guanine nucleotide exchange factor that promotes Ras activation, is a recurrent site of retroviral integrations in *Kras*^*WT*^ leukemias [19, 29]. WES also identified mutations in five genes encoding protein components of the Ras/PI3K pathway in six additional T-ALLs. The low frequency of somatic Ras/PI3K pathway abnormalities in *Kras*^*G12D*^ T-ALLs suggests that expression of endogenous oncogenic *Kras*^*G12D*^ in leukemia initiating cells is sufficient to confer a clonal growth advantage in the absence of additional signaling mutations. Interestingly, however, mutant *Kras* allele frequencies were above 80% in 12 of 15 *Kras*^*G12D*^ leukemias (**Fig 1C**). We considered two potential explanations for this observation: a structural deletion of the WT *Kras* allele or somatic uniparental disomy (UPD) resulting in loss of WT *Kras* and duplication of *Kras*^*G12D*^. The presence of neutral *Kras* copy number inferred from WES data combined with frequent loss of C57Bl/6-specific single nucleotide polymorphisms (SNPs) around the *Kras* locus strongly implicated copy neutral loss of heterozygosity with increased *Kras*^*G12D*^ dosage as the underlying genetic mechanism (**S1 Fig**). These data are indicative of substantial selective pressure favoring the outgrowth of leukemic clones that have increased Ras signaling through a genetic mechanism that precisely duplicates *Kras*^*G12D*^ copy number, and are consistent with recent studies of mouse and human cancers [30, 31]. The overall proportion of mouse leukemias with Ras/PI3K mutations is substantially higher in this model system than in human T-ALL due to the presence of a *Kras*^*G12D*^ knock in mutation in 15 of the 21 of them (**Fig 1B**).

### Many somatic alterations occur in hotspots and are predicated to be pathogenic

**Fig 2:**
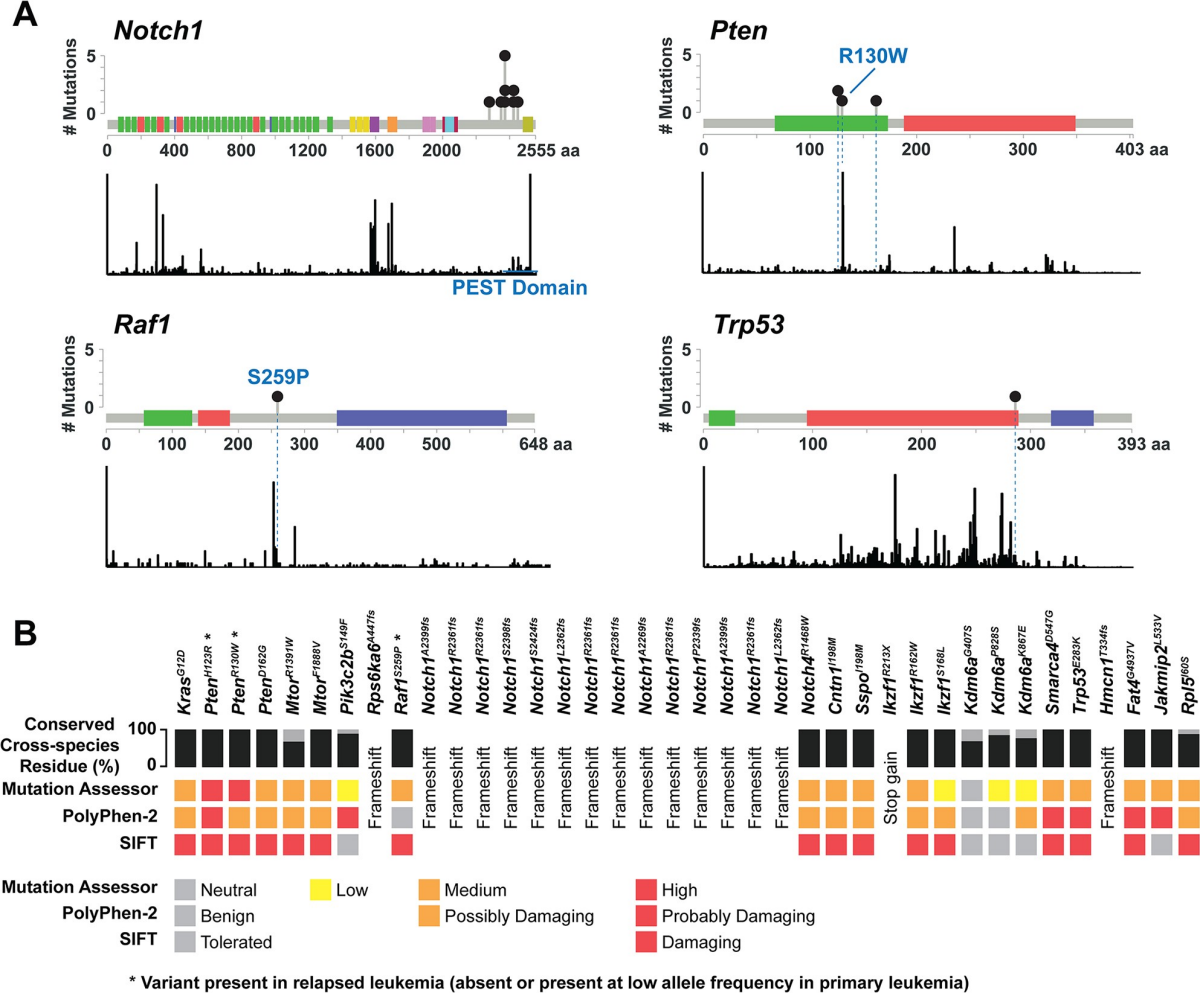
Somatic alterations are frequently predicted to be pathogenic and correspond to mutations in human T-ALL. (**A**) *Notch1*, *Pten*, *Raf1*, and *Trp53* mutations in IM-induced T-ALLs (top panels) occur within hotspot locations that correspond to recurrent mutations in human cancer (bottom panels). (**B**) *In silico* protein function prediction indicates that mutations highlighted in Fig 1 are frequently deleterious and occur at residues that are conserved across multiple species.

To assess the functional consequence of single nucleotide variants (SNVs) and indels in murine T-ALLs, we mapped mutations to the corresponding human protein homologs and compared these data to somatic alterations reported previously. This analysis revealed alterations within conserved functional motifs, including *Pten*^*R130W*^ and *Raf1*^*S259P*^ (**Fig 2A**) [32–34]. *In silico* protein function prediction analysis revealed that most of the reported somatic alterations shown in Fig 1B are expected to abrogate or impair protein function (**Fig 2B**).

### The transcriptional signatures of IM-induced T-ALL closely resemble human T-ALL

**Fig 3:**
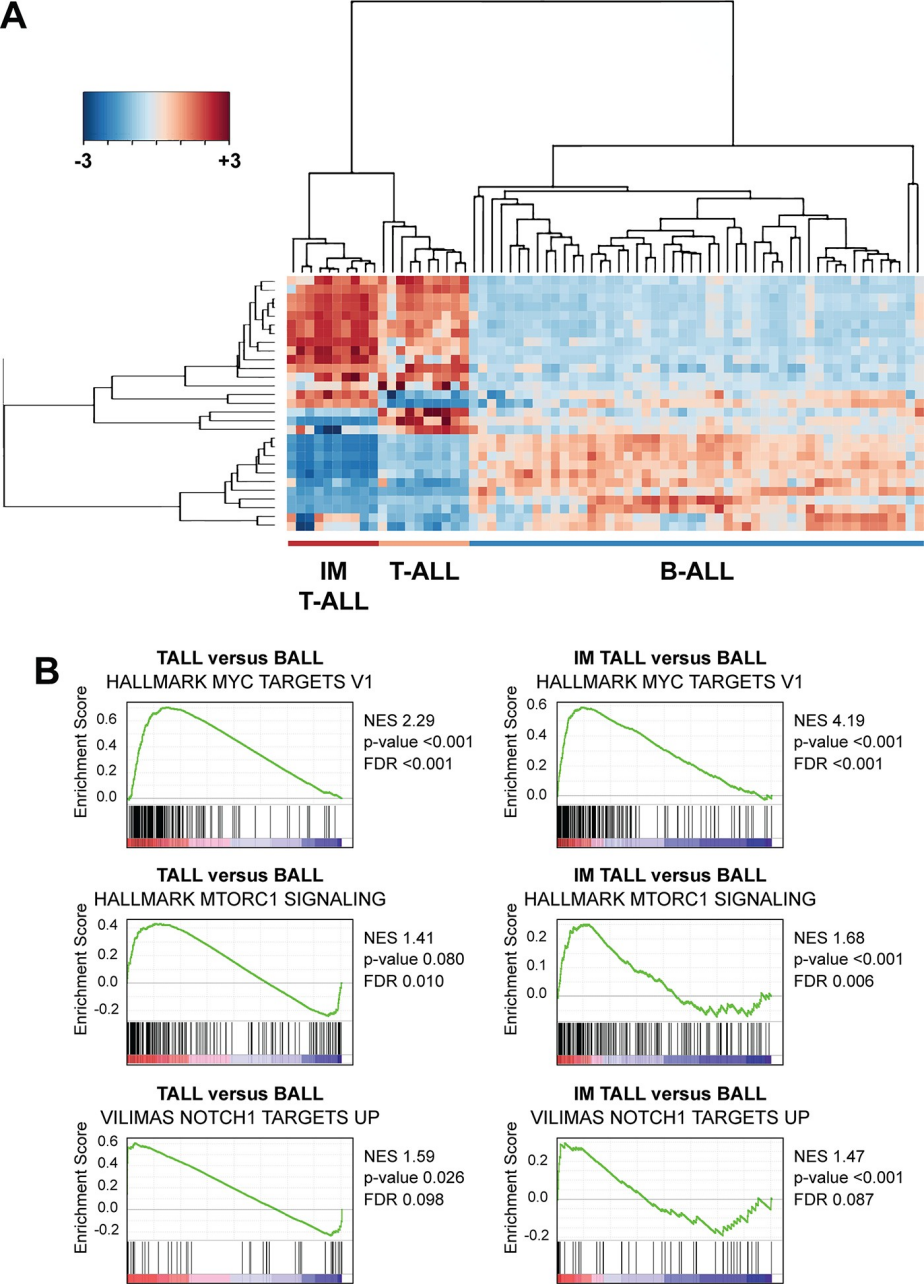
The transcriptional signature and pathway analysis of IM-induced T-ALL cluster with human T-ALL. (**A**) Expression profiling that discriminate human T-ALL from B-ALL clusters IM T-ALL closely with human T-ALL. (**B**) MYC, PI3K, and NOTCH1 pathway gene sets are significantly enriched in murine IM-induced and human T-ALLs compared to B-ALL.

Based on the genetic similarities between human T-ALL and IM-induced T-ALL, we performed RNA-seq to assess the transcriptional landscape of 5 *Kras*^*G12D*^ and 4 *Kras*^*WT*^ T-ALLs. Gene expression signatures that reflect a T lymphoblastic immunophenotype were enriched across these samples, and we observed low-level expression of genes associated with B lymphoblastic and myeloid leukemia immunophenotypes (**S2 Fig**). Consistent with human T-ALL, we also detected distinct and clonal T-cell receptor gene VDJ rearrangements in individual leukemias (**S2 Fig**) [35]. We then compared the gene expression of IM-induced leukemias with previously reported microarray data used to discriminate human T-ALL from various subtypes of B lineage acute lymphoblastic leukemia (B-ALL) [36, 37]. Expression profile signatures that most accurately distinguish T-ALL from B-ALL were used to compare the relative expression in IM-induced T-ALLs, which clustered with human T-ALL (**Fig 3A**). We also performed unbiased gene set enrichment analysis (GSEA) using the Molecular Signature Database Hallmark Gene Sets and C2 Curated Gene Sets to compare: (1) T-ALL versus B-ALL; and, (2) IM-induced T-ALL versus B-ALL. Genes that discriminate various subtypes of B-ALL from each other were included in the analysis [36, 37]. MYC, PI3K, NOTCH1, and TCR pathway gene sets were enriched across human and mouse T-ALLs (**Fig 3B** and **S3A Fig**), and we also observed negative enrichment scores for gene sets that are upregulated in subtypes of B-ALL (**S3B Fig**).

### Clonal evolution occurs at the level of somatic alterations in response to targeted therapies

**Fig 4:**
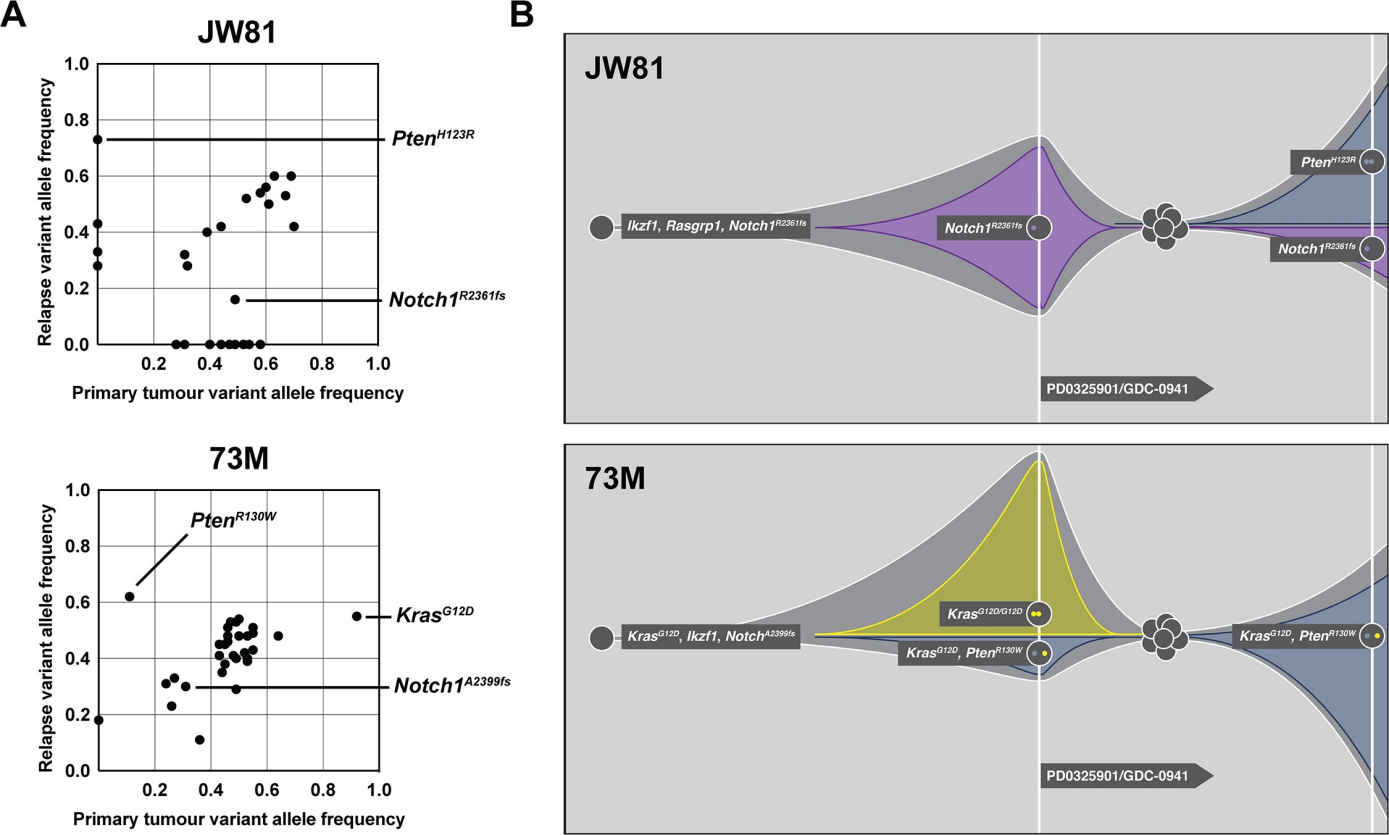
Clonal evolution following combination treatment with PI3K and MEK inhibitors. Leukemias were transplanted and treated with GDC-0941 and PD901. (**A**) Variant allele frequencies of primary versus relapsed leukemias were determined based on whole exome sequencing. Treatment resulted in the emergence of a resistant clone that acquired a *de novo Pten* mutation in T-ALL JW81 and the outgrowth of a subclone of T-ALL 73M with a pre-existing *Pten* mutation at the cost of other drivers. (**B**) Graphical representation of clonal evolution of each primary/relapsed leukemia pair.

Treatment with the pan-PI3K inhibitor GDC-0941 alone and in combination with the MEK inhibitor PD0325901 (PD901) markedly extended the survival of recipient mice transplanted with *Kras*^*WT*^ and *Kras*^*G12D*^ T-ALLs [20], but these leukemias invariably relapsed despite continuous drug administration. As in relapsed human leukemias [38], these resistant murine T-ALLs were derived from pre-existing ancestral clones and they frequently showed loss of activated Notch1 expression and PI3K pathway activation [20]. To identify additional genetic alterations that might contribute to treatment resistance, we performed WES on parental/relapsed T-ALL pairs. This analysis revealed SNVs and indels that were enriched or depleted in individual relapsed leukemias, including two in which inactivating *Pten* mutations emerged at the cost of other drivers (**Fig 4**). In *Kras*^*WT*^ T-ALL JW81, treatment with GDC-0941 and with the combination of GDC-0941/PD901 significantly prolonged survival. At relapse, this leukemia acquired a *de novo Pten*^*H123R*^ mutation and also showed markedly decreased *Notch1* mutant allele frequency (**Fig 4**). Conversely, *Kras*^*G12D*^ T-ALL 73M, which harbored a loss of the WT *Kras* allele and a duplication of *Kras*^*G12D*^, had a minimal response to treatment. Remarkably, however, this leukemia exhibited rapid clonal evolution upon *in vivo* drug exposure with outgrowth of cells showing decreased mutant *Kras*^*G12D*^ allele frequency and substantial enrichment of a pre-existing, low frequency *Pten*^*R130W*^ mutation (**Fig 4**) [32]. Consistent with these results, analysis of heterozygous SNPs revealed enrichment in the 129Sv/Jae SNP allele frequency across the chromosome containing the *Pten* mutant allele and the presence of both WT and mutant *Kras* associated SNPs in the resistant clone (**S4 Fig**).

## Discussion

We describe the first comprehensive molecular characterization of a mouse cancer model initiated by insertional mutagenesis. Through integrated analyses, we demonstrate that T-ALLs initiated by insertional mutagenesis harbor extensive genomic diversity and also acquire a similar spectrum and overall burden of somatic mutations as the corresponding human leukemias. Specifically, many driver genes that are mutated in human T-ALL [39] are also recurrently altered in IM-induced T-ALL, including *Notch1*, *Pten*, *Myb*, and *Ikzf1*. Data from both species implicate deregulated transcriptional programs in disease initiation with subsequent Ras/PI3K and Notch pathway mutations cooperating in leukemic transformation [19, 26, 39].

Genome-wide analysis of solid cancers arising in GEM models expressing one or more oncogenic driver mutations has consistently revealed markedly fewer pathogenic point mutations than in the corresponding human tumors and a higher frequency of large chromosomal alterations [4–8]. This likely reflects the ability of alterations in potent cancer genes such as *Kras*, *Trp53*, *Rb*, and *Pten* to bypass the need for environmentally-induced and age-related mutations that contribute to tumorigenesis. For example, genetic models of *Kras*-driven lung cancer and skin squamous cell carcinoma acquired an average of six and four additional somatic alterations, respectively [4, 6]. By contrast, in parallel analyses, carcinogen-induced models of the same two cancer types acquired an average of 192 and 846 somatic alterations, respectively, with mutational profiles that more accurately reflect human carcinomas [4, 6].

Acute leukemias are distinct from non-hematopoietic cancers with respect to a lower overall somatic mutational burden [25] and also exhibit characteristic patterns of initiating and cooperating events. Deep sequencing studies have identified epigenetic and transcription factor alterations as early events in both lymphoblastic and myeloid acute leukemias, with subsequent signal transduction pathway mutations playing a central role in driving leukemic outgrowth [26, 38, 40, 41]. Molecular analysis of IM-induced T-ALLs suggest that they recapitulate this pathogenic sequence with disease-initiating retroviral integrations that deregulate transcriptional programs generating preleukemic clones [19], that subsequently acquired an average of 13 additional somatic alterations, including recurring mutations in Ras/PI3K pathway genes in *Kras*^*WT*^ leukemias. This latter observation is consistent with the fact that activating *Kras*^*G12D*^ expression as a secondary event increased leukemia penetrance and also reduced latency [19]. In addition, resistant leukemias that emerged during *in vivo* treatment with MEK and PI3K inhibitors invariably retained multiple retroviral integrations found in the corresponding drug-sensitive parental T-ALL, but frequently showed loss of *Notch1* mutation [20].

Frequent uniparental disomy resulting in loss of WT *Kras* and duplication of *Kras*^*G12D*^ in 14 of 17 IM-induced T-ALLs is consistent with recent work demonstrating that *KRAS* mutations in acute myeloid leukemia and colorectal cancer frequently exhibit allelic imbalance that modulate competitive fitness and MEK dependency [30]. Similarly, a comprehensive genomic characterization of 264 primary T-ALLs showed that *KRAS* mutations, but not *NRAS* mutations, were specifically associated with mutant allele fractions of greater than 0.7 in a subset of patients at diagnosis [39]. Another study that investigated large numbers of murine *Kras*^*G12D*^ pancreatic and lung cancers also identified frequent *Kras* allelic imbalance that was modulated by treatment with a MEK inhibitor [8]. The panel of primary mouse leukemias described here are novel reagents for investigating the effects of restoring WT *Kras* expression on fitness and for interrogating K-Ras protein dimerization and other candidate molecular mechanisms of growth inhibition [42, 43].

*MYC* is a downstream target of activated Notch1 signaling that contributes to T-ALL pathogenesis [44–47]. As the mouse *Myc* gene is located on chromosome 15, it is intriguing that 13 of 21 T-ALLs analyzed in this study exhibited trisomy 15. However, *Myc* expression was similar in leukemias with and without trisomy 15 and we did not observe any with focal *Myc* amplification. Similarly, Mullighan, et al. [39] identified 12 human T-ALLs with trisomy 8 or large gains of 8q, which is the chromosome arm that contains *MYC*, that were not characterized by elevated *MYC* expression and did not harbor focal *MYC* amplification. Thus, the role of *MYC/Myc* in promoting the outgrowth of T-ALLs with trisomy 8 (human) or 15 (mouse) is uncertain.

*NRAS*, *KRAS*, *PTEN*, and *AKT* mutations are associated with worse outcomes in patients newly diagnosed with T-ALL [39, 48, 49], are enriched in relapse specimens [38, 50], and have previously been shown to provide a proliferative advantage based on xenograft models [51]. While cure rates have improved substantially in T-ALL, relapsed disease is still associated with dismal outcomes [52]. Furthermore, whereas T-ALLs characterized by activated Notch signaling are vulnerable to inhibitors of the Notch processing enzyme gamma secretase, *Pten* inactivation overcomes this dependency [53]. This might, in part, underlie the disappointing response rates seen in early phase clinical trials of agents targeting gamma secretase in T-ALL [54, 55]. Together, these data indicate a need for both therapeutic approaches directed against hyperactive Ras/PI3K signaling in T-ALL and for robust preclinical models to test them. Along these lines, it is notable that the 21 primary leukemias characterized here exhibit frequent alterations in Ras and PI3K pathway genes, some of which were enriched at relapse. This panel of transplantable primary and relapsed/refractory leukemias with known genetic aberrations thus comprises a unique resource for testing novel therapeutic strategies for high-risk subsets of T-ALL and for delineating additional resistance mechanisms.

## Materials and methods

### Ethics statement

All animal experiments conformed to national regulatory standards and were approved by the University of California, San Francisco Committee on Animal Research (IACUC Protocol Approval Number AN136527).

### Murine leukemias

All animal experiments conformed to national regulatory standards and were approved by the University of California, San Francisco Committee on Animal Research. C57Bl/6 x 129Sv/Jae *Mx1-Cre* x *Kras*^*LSL-G12D*^ mice (*Kras*^*G12D*^) and wild-type littermates (*Kras*^*WT*^) were injected with MOL4070LTR at 3–5 days of age and *Kras*^*G12D*^ expression was activated at 21 days of age by administering a single dose of polyinosinic-polycytidylic acid (pIpC) as previously described [19, 20]. For adoptive transfer of acute leukemias, 2 × 10^6^ cells were injected retro-orbitally into 8–12 week old wild-type C57Bl/6 x 129Sv/Jae F1 recipient mice that had been irradiated with 450 cGy. Mice that appeared ill were euthanized and underwent full pathological examination. Flushed bone marrow cells were isolated to confirm relapse and to assess for tumor purity. Relapsed leukemia cells were cryopreserved for molecular and genetic analysis.

### Whole exome sequencing

Genomic DNA was extracted from the bone marrows of mice transplanted with IM-induced leukemias and then sheared to generate 150 to 200 base pair fragments using a Covaris S2 focused-ultrasonicator. Indexed libraries were prepared using the Agilent SureSelect XT2 Reagent Kit for the HiSeq platform. Exomes were captured using the Agilent SureSelect XT2 Mouse All Exon bait library. Sample quality and quantity were assessed using the Agilent 2100 Bioanalyzer instrument. Paired-end 100 base pair reads were generated on an Illumina HiSeq 2000 platform. All sequence data including read alignment; quality and performance metrics; post-processing, somatic mutation and DNA copy number alteration detection; and variant annotation were performed as previously described [56, 57] using the mm10 build of the mouse genome. Briefly, reads were aligned with Burrows-Wheeler Aligner [58], and processed using Picard (http://broadinstitute.github.io/picard) tools and the Genome Analysis Toolkit (GATK) [59] to perform base quality recalibration and multiple sequence realignment. Single nucleotide variants and indels were detected with the MuTect [60] and Pindel [61] algorithms, respectively. Candidate somatic mutations were manually reviewed using Integrative Genomics Viewer [62] and were confirmed by performing orthogonal Sanger sequencing validation. Tumor-specific copy number alterations were inferred from depth of coverage and B allele frequencies using control normal bone marrow samples. Loss of heterozygosity analysis was performed using GATK UnifiedGenotyper to compare germline heterozygous SNPs that differ between C57Bl/6 and 129Sv/Jae mouse strains called at a depth of 20x or greater between leukemia and control bone marrow samples.

### MOL4070 integration cloning

Restriction enzyme digestion of genomic DNA from mouse T-ALLs, gel electrophoresis, Southern blot analysis, and hybridization with a MOL4070LTR-specific probe was performed as described previously [20, 30]. Junctional fragments at sites of retroviral integration were identified as previously described using linker-based PCR amplification and sequencing [63, 64].

### Mutation mapping and in silico protein function prediction

Mutations were mapped onto corresponding protein sequence using MutationMapper [65]. Associated mutational frequencies in human cancer were extracted from Catalogue of Somatic Mutations in Cancer (COSMIC, version 82) [66]. Pairwise alignments between homologous proteins were generated using NCBI HomoloGene and BLASTP. *In silico* protein function prediction was performed using Mutation Assessor [67], PolyPhen-2 [68], and Sorted Intolerant From Tolerant (SIFT) [69].

### Transcriptome sequencing

Total RNA was extracted from the bone marrows of mice transplanted with IM-induced leukemias using TRIzol. Phenol-chloroform-isoamyl extraction and ethanol precipitation were performed. Total RNA (1 μg) was treated with DNase I at room temperature for 15 min. The integrity of the DNase I-treated RNA was analyzed on an Agilent 2100 Bioanalyzer before selection of poly(A) mRNA. Selection for poly(A) mRNA and subsequent cDNA synthesis and library preparation were carried out using the Illumina TruSeq RNA sample preparation kit according to the manufacturer's protocol. cDNA was fragmented (200-bp peak) with a Covaris E210 ultrasonicator prior to library preparation. The quality and size of the final library preparation were analyzed on an Agilent 2100 Bioanalyzer. Paired-end 100 base pair reads were generated on an Illumina HiSeq 2500 platform. Reads were aligned with Bowtie 2 [70] using the mm10 database, and transcript quantification was performed with RNA-seq by Expectation Maximization (RSEM) [71].

### Gene expression analysis

Raw microarray gene expression profiling data on patients with T-ALL and B-ALL was downloaded from publicly accessible repositories as previously described [36, 37]. Microarray data was normalized using the robust multi-array average (RMA) method [72]. Probe identifiers were mapped to associated gene identifiers using the Affymetrix HG U95A Annotations Reference (Release 36). Gene identifiers generated from RNA-seq data on IM-induced T-ALLs were mapped to human orthologs using NCBI HomoloGene. FPKM values were normalized to the microarray data using the trimmed mean of M-values (TMM) method [73]. Normalization was assessed by plotting average versus fold change normalized expression (MA plot) between the two datasets and average normalized expression comparing the two datasets. Clustering and heatmap dendrogram analysis was performed using a correlation similarity metric and average linkage clustering as previously described [74]. Gene set enrichment analysis was performed as previously described [75]. Gene set permutation was performed to calculate p-values and FDR due to small sample size.

## Supporting information

S1 Fig Frequent copy neutral loss of heterozygosity at the *Kras* locus with duplication of the mutant *Kras^G12D^* allele. Leukemias were generated on a C57Bl/6 x 129Sv/Jae F1 strain background allowing us to analyze single nucleotide polymorphisms (SNPs) that differ between the two strains. Raw SNP allele frequencies are plotted against relative position on chromosome 6. The location of *Kras* is indicated with a diamond. *Kras* mutant IM T-ALLs frequently have loss of heterozygosity (AF = allele frequency), which is copy neutral (LR = log ratio copy number) and does not occur in wild-type *Kras* counterparts.
(TIF)

S2 Fig IM-induced T-ALL antigen expression is highly similar to human T-ALL patterns of expression. RNA-seq was performed on 9 primary IM-induced T-ALLs. The gene expression of IM T-ALLs is lineage specific with high gene expression of T-ALL specific antigens and low expression of B-ALL and/or myeloid specific antigens. TCR gene rearrangements are variable based on the primary leukemia (gray).
(EPS)

S3 Fig IM-induced T-ALL and human T-ALL have similar pathway enrichment compared to B-ALL. (**A**) A TCR pathway gene set are significantly enriched in murine IM-induced and human T-ALLs compared to B-ALL. (**B**) Gene sets associated with various subtypes of B-ALL are enriched in the respective subtype of B-ALL compared to either T-ALL or IM-induced T-ALL.
(EPS)

S4 Fig Clonal evolution following treatment with combination MEK and PI3K inhibitors. (**A**) Sequence reads aligned to *Pten* locus generated from WES of JW81 primary (top panel) versus relapsed leukemia (bottom panel). The *Pten* mutation is not detectable within the primary tumor, whereas it is present at a 0.547 (read depth 137) allele frequency after treatment with combination MEK and PI3K inhibitors. (**B**) *Kras*^*G12D*^ allelic frequencies were determined based on Sanger sequencing and relative peak intensities of mutant versus wild-type alleles. Loss of heterozygosity during leukemogenesis is a common event resulting in near loss of wild-type *Kras* in multiple independent leukemias. Outlier leukemia T-ALL 73M initially lost WT *Kras*, but re-acquired *Kras*^*G12D*^ heterozygosity following treatment with combination MEK and PI3K inhibitors (red line). (**C**) Sequence reads aligned to *Kras* and *Pten* locus generated from WES of 73M primary versus relapsed leukemias. The *Kras* allele frequency decreases from 0.920 (read depth 138) to 0.547 (read depth 150), whereas the *Pten* allele frequency increases from 0.108 (read depth 102) to 0.618 (read depth 137) after treatment with combination MEK and PI3K inhibitors. (**D**) SNP allele frequencies plotted against relative position on chromosome 6 (*Kras*) and 19 (*Pten*) for parental and relapsed 73M and (**E**) copy neutral number for both parental and relapsed 73M support uniparental disomy as the underlying mechanism for increased mutant *Kras* and *Pten* allele frequencies.
(TIF)
