# Supplementary figures and images for: Convergent genetic aberrations in murine and human T lineage acute lymphoblastic leukemias
Source: PLoS Genet. 2019 Jun 14;15(6):e1008168. doi: 10.1371/journal.pgen.1008168 (PMC6594654; doi:10.1371/journal.pgen.1008168)

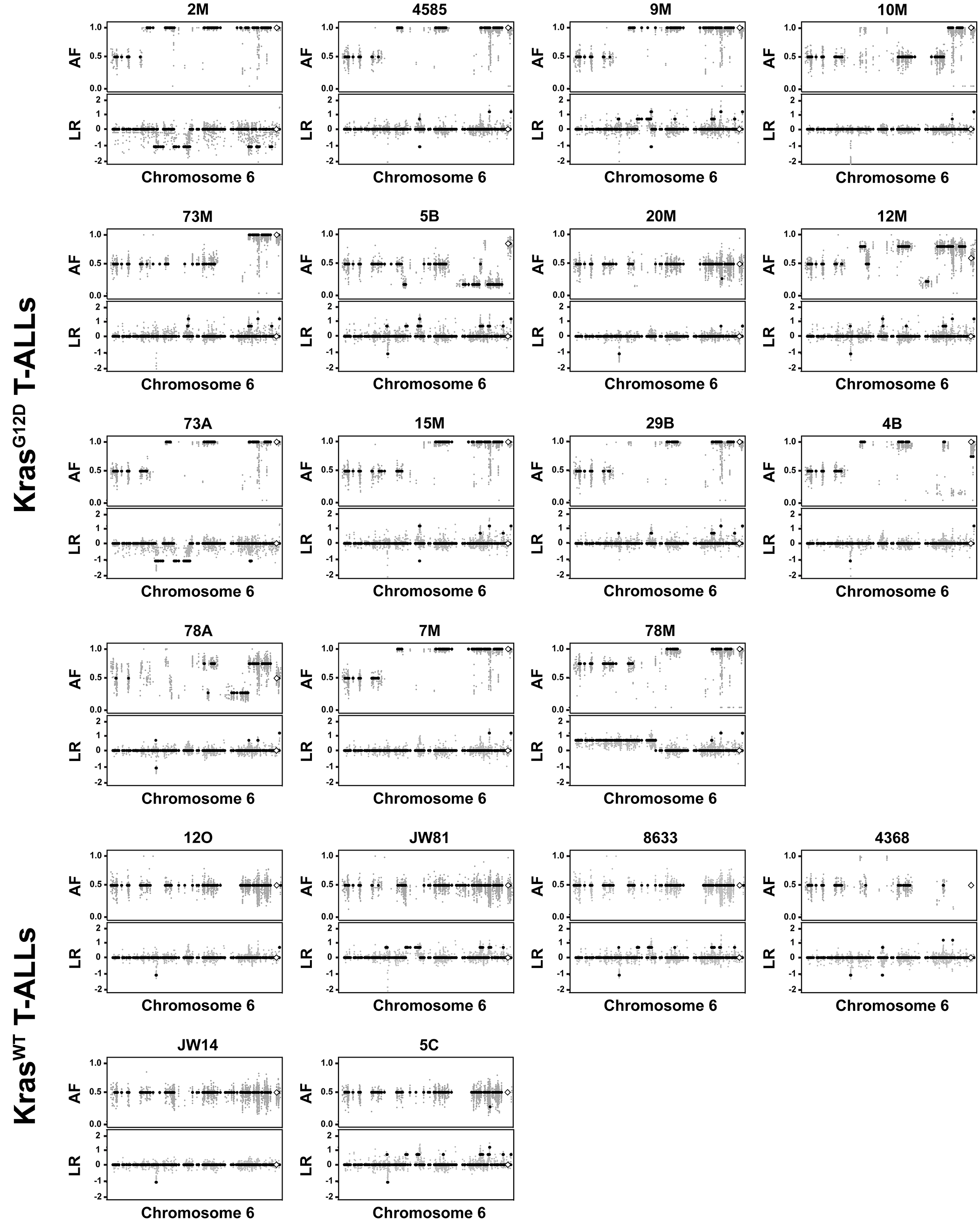

Supplement: S1 Fig — Leukemias were generated on a C57Bl/6 x 129Sv/Jae F1 strain background allowing us to analyze single nucleotide polymorphisms (SNPs) that differ between the two strains. Raw SNP allele frequencies are plotted against relative position on chromosome 6. The location of Kras is indicated with a diamond. Kras mutant IM T-ALLs frequently have loss of heterozygosity (AF = allele frequency), which is copy neutral (LR = log ratio copy number) and does not occur in wild-type Kras counterparts. (TIF) [file pgen.1008168.s001.tif]

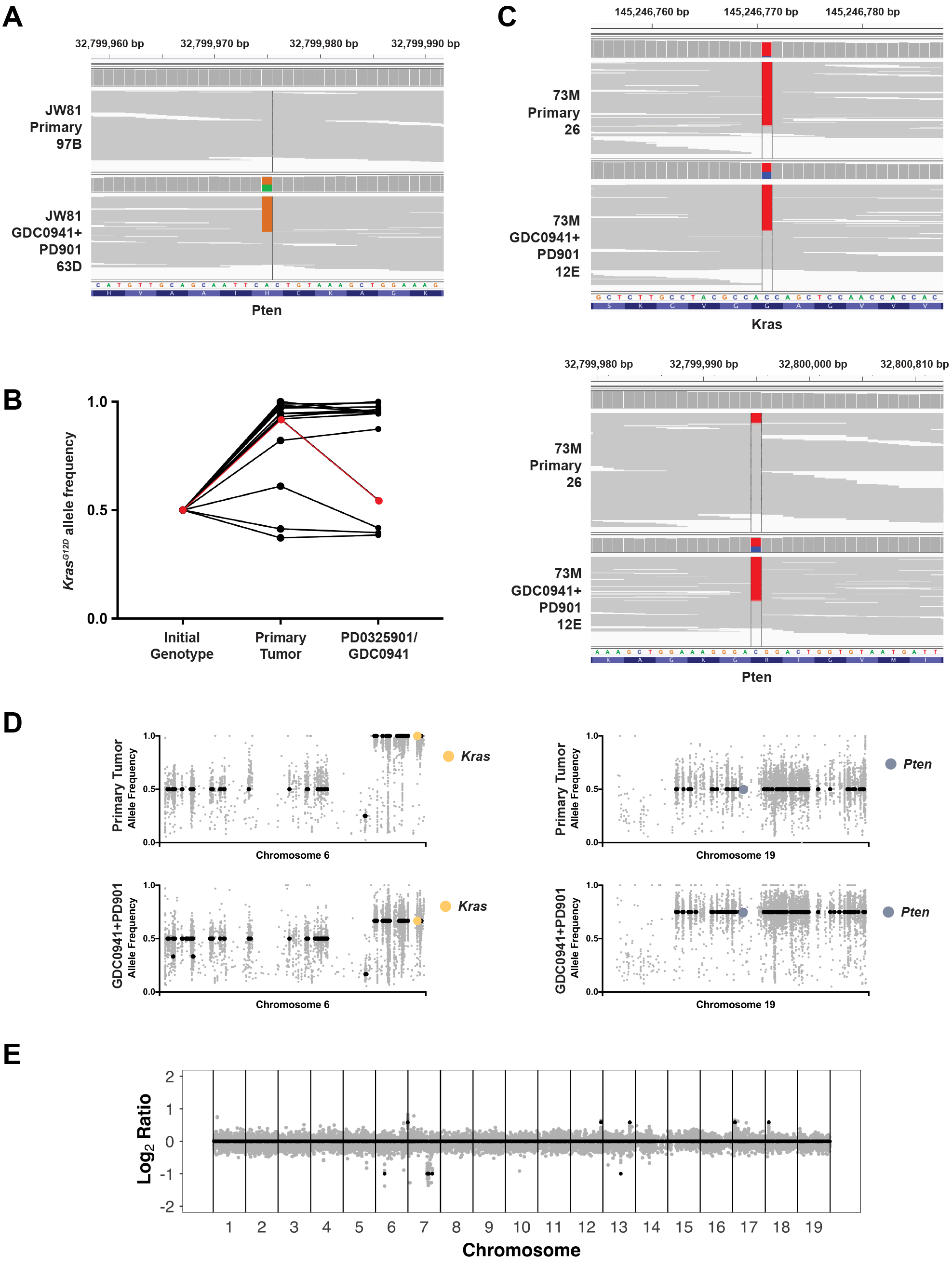

Supplement: S4 Fig — (A) Sequence reads aligned to Pten locus generated from WES of JW81 primary (top panel) versus relapsed leukemia (bottom panel). The Pten mutation is not detectable within the primary tumor, whereas it is present at a 0.547 (read depth 137) allele frequency after treatment with combination MEK and PI3K inhibitors. (B) KrasG12D allelic frequencies were determined based on Sanger sequencing and relative peak intensities of mutant versus wild-type alleles. Loss of heterozygosity during leukemogenesis is a common event resulting in near loss of wild-type Kras in multiple independent leukemias. Outlier leukemia T-ALL 73M initially lost WT Kras, but re-acquired KrasG12D heterozygosity following treatment with combination MEK and PI3K inhibitors (red line). (C) Sequence reads aligned to Kras and Pten locus generated from WES of 73M primary versus relapsed leukemias. The Kras allele frequency decreases from 0.920 (read depth 138) to 0.547 (read depth 150), whereas the Pten allele frequency increases from 0.108 (read depth 102) to 0.618 (read depth 137) after treatment with combination MEK and PI3K inhibitors. (D) SNP allele frequencies plotted against relative position on chromosome 6 (Kras) and 19 (Pten) for parental and relapsed 73M and (E) copy neutral number for both parental and relapsed 73M support uniparental disomy as the underlying mechanism for increased mutant Kras and Pten allele frequencies. (TIF) [file pgen.1008168.s004.tif]
